# Complete chloroplast genomes of *E. umbellata* Thunb., *E. multiflora* Thunb., *E. macrophylla* Thunb., and *E. glabra* Thunb. (Elaeagnaceae)

**DOI:** 10.1080/23802359.2020.1779142

**Published:** 2020-06-17

**Authors:** Yonguk Kim, Jawon Shin, Dong-Wook Kim, Hak-Sung Lee, Chulyung Choi

**Affiliations:** a Jeonnam Institute of Natural Resources Research, Jeollanam-do, Republic of Korea; b Coscience Co. Ltd, Jeollanam-do, Republic of Korea

**Keywords:** *Elaeagnus umbellata*, *Elaeagnus multiflora*, *Elaeagnus macrophylla*, *Elaeagnus glabra*, complete chloroplast genome, Elaeagnaceae, phylogenetic analysis

## Abstract

*Elaeagnus* is a genus which consists about 70 species of flowering plants in the family Elaeagnaceae, and its edible fruit is a natural product used as food and in traditional medicine. In this study, we sequenced the complete chloroplast (cp) genome of four species, namely *Elaeagnus umbellate* Thunb., *E. multiflora* Thunb., *E. macrophylla* Thunb., and *E. glabra* Thunb., to study their phylogenetic relationships within the Elaeagnaceae. Total lengths of the chloroplast genome were 152,261 bp, 152,267 bp, 152,224 bp, and 152,227 bp, respectively. The four genomes had representative quadripartite structures, with an LSC region (82,207 bp, 82,191 bp, 82,136 bp, and 82,139 bp) and an SSC region (18,262 bp, 18,282 bp,and 18,278 bp for both species) separated by a pair of IRs (25,896 bp, 25,897 bp, and 25,905 bp for the latter two species), respectively. Moreover, they were composed of 136–137 genes, including 88 protein-coding genes, 40–41 tRNA genes, and 8 rRNA genes. A maximum likelihood phylogenetic analysis indicated that *E. umbellata* was most closely related to *E. multiflora*, whereas *E. macrophylla* was close to *E. glabra*.

The genus *Elaeagnus* of the family Elaeagnaceae are plants that are distributed worldwide but are native mainly to temperate and subtropical regions of Asia, North America, southeastern Europe and Australia (Patel 2015). Many *Elaeagnus* species are used in health food and folk medicine. Among these, *E. umbellata*, *E. multiflora*, *E. macrophylla*, and *E. glabra*are are widely distributed in Korea and commonly consumed as food and herbal medicine (Shin et al. 2008; Li et al. 2009; Kim et al. 2016). Most research on *Elaeagnus* has focused on the aspects of commercial and industrial applications, such as their chemical and nutritional compositions. Studies on the molecular genetic diversity in the *Elaeagnus* genus are scarce. In this study, we used the Illumina Hiseq X platform to sequence the complete chloroplast (cp) genomes of four species: *E. umbellata*, *E. multiflora*, *E. macrophylla*, and *E. glabra*.

Fresh leaf material from four species were collected from Naju-si (35°00′22.0ʺN 126°49′27.8ʺE) in Jeollanam-do (Province), Korea. The specimens were deposited at the herbarium of the Jeollanam-do Forest Resources Institute (JFRI), Korea (specimen code JFRI00342741, JFR00342742, JFR00342743, and JFR00342744, respectively).

Total genomic DNA wa sextracted from fresh leaves by the CTAB method, and SEEDERS Inc. (Daejeon, Korea) performed whole-genome resequencing with the Illumina HiseqX Ten platform. Raw reads were filtered using Dynamic Trim and Length Short of the SolexaQA (ver.1.13) package (Cox et al. 2010).

Clean reads were first aligned to the cp genome of *E. macrophylla* (GenBank Accession No. NC_028066). Filtered reads were assembled into contigs in the software NOVOPlastyv 2.7.1 (Dierckxsens et al. 2017) and Fast-Plast v1.2.8 (Mckain et al. 2018). The physical maps of the new chloroplast genomes were generated using OGDRAWv 1.3.1 (Greiner et al. 2019). Finally, the validated complete cp genome sequences were submitted to the GenBank with accession numbers LC522506, LC523635, LC522136, and LC522137, respectively.

Total lengths of the chloroplast genome sequences of *E. umbellata*, *E. multiflora*, *E. macrophylla*, and *E. glabra* were 152,261 bp, 152,267 bp, 152,224 bp, and 152,227 bp, respectively. For *E. umbellata*, a pair of inverted repeats (IRs) of 25,896 bp was separated by a small single-copy (SSC) region of 18,262 bp and a large single-copy (LSC) region of 82,207 bp. The *E. multiflora* cp genome consisted of a LSC region of 82,191 bp, a SSC region of 18,282 bp, and IRs of 25,897 bp. *Elaeagnus Macrophylla* contained a LSC region of 82,136 bp, a SSC region of 18,278 bp, and IRs of 25,905 bp.The *E. glabra* cp genome consisted of a LSC region of 82,139 bp, a SSC region of 18,278 bp, and IRs of 25,905 bp. These new sequences each had a total of 137 genes (except for *E. umbellata* with 136 genes), including 88 protein-coding genes, 41 tRNA genes (except for *E. umbellate* with 40 genes), and 8 rRNA genes. The overall GC content of each whole plastome was 37.1%.

To investigate the phylogeny of *E. umbellata*, *E. multiflora*, *E. macrophylla*, and *E. glabra*, nine complete cp genomes belonging to Elaeagnaceae and related families were downloaded from GenBank and aligned using ClustalW (Thompson et al. 1994). A maximum likelihood method tree based on the Tamura–Nei model was constructed using MEGA 7.0 (Kumar et al. 2016). bThe ML tree analysis showed that *E. umbellata* had a closer relationship with *E. multiflora*; meanwhile, *E. macrophylla* was more closely related to *E. glabra*, with 100% bootstrap support (Figure 1).

**Figure 1.**
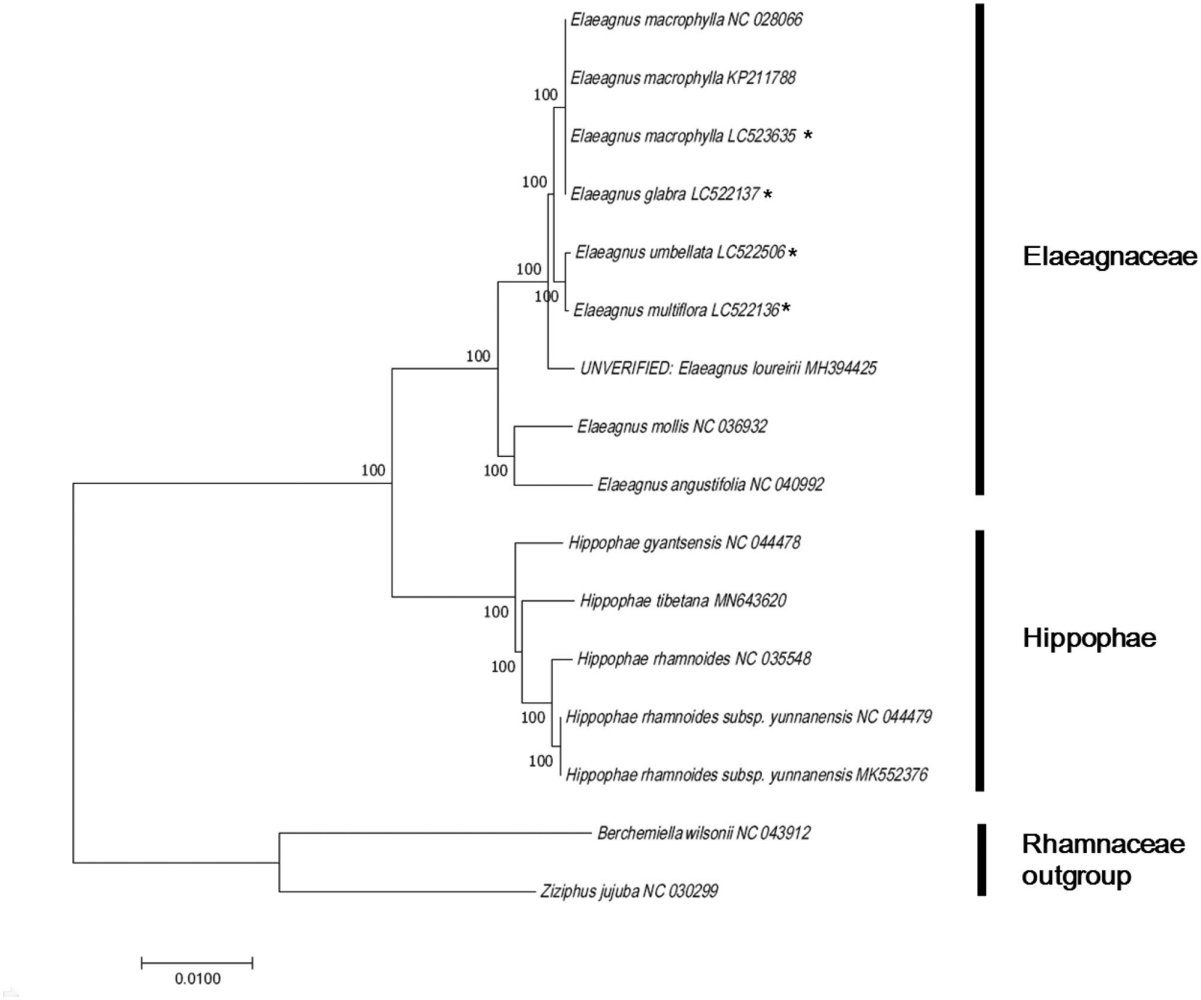
Phylogenetic tree based on 16 complete chloroplast genome sequences of Elaeagnaceae species. All the sequences were downloaded from NCBI GenBank.

## Data Availability

The data that support the findings of this study openly available in National Center for Biotechnology Information (NCBI) at https://www.ncbi.nlm.nih.gov, accession numbers LC522506, LC523635, LC522136, and LC522137, respectively.
